# Kidney Damage in Pediatric Obesity: Insights from an Emerging Perspective

**DOI:** 10.3390/jcm13237025

**Published:** 2024-11-21

**Authors:** Gianmario Forcina, Margherita Luciano, Vittoria Frattolillo, Simona Mori, Noemi Monaco, Stefano Guarino, Pierluigi Marzuillo, Emanuele Miraglia del Giudice, Anna Di Sessa

**Affiliations:** Department of Woman, Child, and General and Specialized Surgery, University of Campania “Luigi Vanvitelli”, 80138 Naples, Italy; gianmario.forcina@gmail.com (G.F.); margherita.luciano2@gmail.com (M.L.); vitto.fratt@gmail.com (V.F.); esse.mori@gmail.com (S.M.); monaconoemi1@gmail.com (N.M.); stefano.guarino@policliniconapoli.it (S.G.); emanuele.miraglia@unicampania.it (E.M.d.G.); anna.disessa@unicampania.it (A.D.S.)

**Keywords:** kidney damage, children, obesity, cardiometabolic risk, management

## Abstract

The role of obesity as a risk factor for chronic kidney disease (CKD) in adulthood has been well established. Over the last years, kidney damage (KD) has emerged as a significant consequence of obesity since childhood. Indeed, a complex interplay of metabolic factors, including insulin resistance (IR), hypertension, oxidative stress, adipose tissue dysfunction, and systemic inflammation, might affect renal hemodynamics, contributing to CKD development over time in at-risk young patients. As the prevalence of pediatric obesity continues to rise globally, understanding the implications for kidney health in terms of early intervention is of paramount importance. Careful monitoring of kidney function within a multidisciplinary approach in children with obesity is crucial for detecting early KD, allowing for timely lifestyle modifications and treatment. In this framework, continued research is essential to further elucidate mechanisms linking obesity and KD and to explore not only effective preventive strategies but also the long-term impact of obesity on kidney health in children with obesity. Given the intimate link of KD with the metabolic milieu in children with obesity, we aimed to provide a comprehensive and insightful overview on KD and its implications in pediatric obesity by reviewing the most recent literature in the field.

## 1. Introduction

In recent years, kidney damage (KD) has garnered significant scientific interest as a consequence of obesity since childhood [1,2,3]. Obesity is defined as having a body mass index (BMI) greater than or equal to the 95th percentile for age and gender according to growth reference charts in children aged ≥2 years [4].

While the role of obesity as a risk factor for chronic kidney disease (CKD) in adulthood has been well documented [4,5,6,7], emerging evidence has also linked KD to pediatric obesity [2,8,9]. Robust data indicate that children with obesity face a heightened risk of KD [1,8,10,11,12], exacerbating their already-increased cardiometabolic burden (including insulin resistance [IR], metabolic syndrome, type 2 diabetes [T2D], and metabolic-associated steatotic liver disease [MASLD]) [13,14,15,16].

Defining KD in childhood is still challenging because of the physiological age-related modifications of GFR during the first years of life and the significant variability in clinical presentations [1,2]. KD in children with obesity can be defined by the presence of reduced eGFR and/or albuminuria [17], confirmed over a three-month period [18]. Specifically, reduced eGFR is defined as <90 mL/min/1.73 m^2^, while albuminuria is indicated by an albumin-to-creatinine ratio (ACR) of ≥30 mg/g [13]. To effectively manage children with obesity, detection of hypertension is also essential [19]. Nevertheless, the initially subclinical nature of KD might further complicate its early identification, leading to misdiagnosis and potentially accelerating its progression to CKD [6,7,20].

While CKD has been found to affect >10% of the general adult population [21], it has become a serious health issue also for pediatric patients [21,22,23]. Indeed, in the last decade, pediatric CKD prevalence ranged from 15 to 74.7 cases per million of the age-related population [24], but estimates are currently increasing worldwide with the rapid spread of certain well-known pathophysiological risk factors, such as obesity, IR, and hypertension in childhood [1,2,3].

KD prevalence in children with obesity has been reported at approximately up to 20.9% [1,2,11,25]. However, estimates of KD within this population significantly varied due to the clinical heterogeneity of the studies, which investigated different KD features, including hyperfiltration, microalbuminuria, hypertension, and reduced estimated glomerular filtration rate (eGFR) [1,2,26,27].

The intricate interplay among IR, chronic inflammation, oxidative stress, and adipokine dysregulation creates a detrimental vicious cycle [28,29], serving as the primary pathophysiological mechanism linking KD to obesity and its related dysmetabolism [20,28,29]. This interplay significantly affects renal hemodynamics, contributing to the development of CKD over time [20,29].

Given these insights, KD should be considered a critical component within the broader spectrum of obesity-related consequences [2], as recently underlined by experts [10].

By reviewing the existing literature on KD in children with obesity, we aimed to provide a comprehensive perspective on this compelling research area, emphasizing the crucial importance of kidney health in these young patients, who are intrinsically at greater cardiometabolic risk. A thorough literature search was conducted across several major databases, including PubMed, Medline, Scopus, Web of Science, and Google Scholar, to identify relevant studies published between January 2003 and September 2024. Additionally, reference lists from key articles and systematic reviews were manually searched to capture any studies that might have been missed in the database searches.

Keywords such as “kidney damage”, “children”, “obesity”, “treatment”, “diagnosis”, and “prevention” were used in various combinations. Studies were screened based on predefined inclusion and exclusion criteria. Excluded from the review were studies not published in English, non-peer-reviewed articles, case reports, studies with insufficient data, and unpublished reports. It is important to acknowledge that potential publication bias, stemming from the selective inclusion of studies and exclusion of unpublished data, may compromise the reliability and generalizability of the findings.

## 2. Pathophysiology of KD in Pediatric Obesity

**Figure 1:**
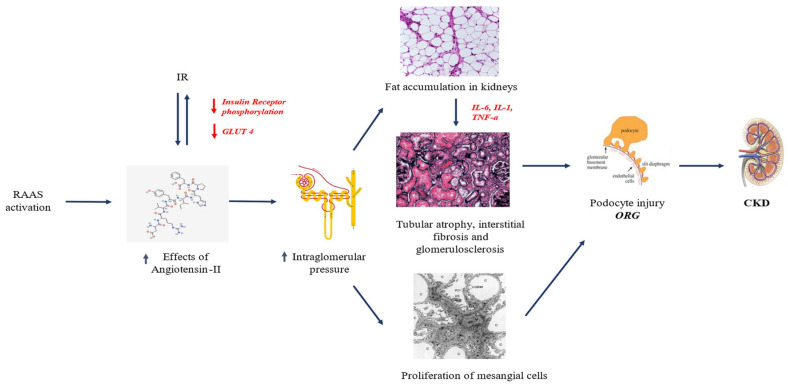
Pathophysiology of KD in pediatric obesity. Abbreviations: CKD, chronic kidney disease; GLUT-4, glucose transporter 4; IL-1, interleukin-1; IL-6, interleukin-6; ORG, obesity-related glomerulopathy; RAAS, renin–angiotensin–aldosterone system; TNF-α, tumor necrosis factor alpha.

Obesity has been well recognized as a major risk factor for pediatric CKD development [2,21,26,30], but its exact pathophysiology remains to be fully elucidated [21,30]. To date, a tangled interplay among different pathophysiological mechanisms has been documented in the pathophysiology of obesity-related KD [21,24,26,28], in which IR, inflammation, and oxidative stress play key roles [2] (Figure 1).

Obesity-related KD has been described as a distinct pathological entity known as obesity-related glomerulopathy (ORG), characterized by glomerulomegaly and progressive glomerulosclerosis, leading to kidney function decline [1,24,29]. Notably, various factors (e.g., IR, renin–angiotensin–aldosterone system [RAAS], renal lipotoxicity, inflammation, and adipokine dysfunction) have been identified as main pathogenic players in this context [2,29,31,32].

In this framework, the negative influence of reduced nephron numbers on growth and long-term kidney function should also be taken into account when assessing the potential for KD [33].

### 2.1. RAAS Upregulation

Obesity is linked to increased metabolic and hemodynamic demands on the kidneys [23,28,29]. This occurs through heightened vascular permeability due to arteriolar vasodilation, which in turn leads to upregulation of RAAS. Consequently, the raised sodium reabsorption in the proximal convoluted tubule (PCT) increases the risk of hypertension [1,26,34] and proteinuria [6,9,21,30]. This risk is also exacerbated by glomerular hyperfiltration due to sodium accumulation, resulting in hemoconcentration in the post-glomerular region [23,31]. Hyperfiltration is closely associated with glomerulomegaly, which can ultimately lead to focal segmental glomerulosclerosis (FSGS) [31]. This mechanism creates a vicious cycle, where glomerular damage promotes further afferent arteriolar vasodilation, which in turn drives efferent arteriolar vasoconstriction [31]. Additionally, angiotensin II, the primary effector of the renin–angiotensin–aldosterone system (RAAS), contributes to a chronic state of inflammation and oxidative stress. It acts both as a growth factor and as a pro-inflammatory cytokine, exacerbating KD [31]. RAAS upregulation, leading to an imbalance between elevated levels of angiotensin II and the ACE2/Ang (1–7) axis, is also linked to deterioration of the metabolic profile, particularly in glucose metabolism. Overexpression of angiotensin II stimulates the production of pro-inflammatory adipokines, which impair the function of glucose transporter 4 (GLUT4) and hinder insulin receptor phosphorylation, further compromising glucose homeostasis [31].

### 2.2. Renal Lipotoxicity

The strong association between childhood obesity and dyslipidemia, driven by pathological adipose tissue accumulation, is well documented [23,32,35]. The metabolic dysfunction resulting from dyslipidemia leads to persistent low-grade inflammation, which in turn promotes excess fat deposition, including in the kidneys. This creates a state of nephrotoxic lipotoxicity [36,37]. The accumulation of fat in the kidneys sets off a vicious cycle, with the secretion of cytokines, growth factors, and pro-inflammatory adipokines that further fuel the ongoing inflammation and oxidative stress [23,32]. These factors collectively trigger mitochondrial oxidative stress, alter the cytoskeleton, and promote IR, leading to a progressively complex state of basal inflammation [32]. This inflammatory cascade contributes to the progression of glomerulosclerosis and, ultimately, renal fibrosis [32]. Notably, fat accumulation in the peri-vascular space of the renal sinus exacerbates endothelial dysfunction and increases vascular permeability [32], further promoting RAAS upregulation. Moreover, dyslipidemia and renal lipotoxicity are closely linked, as damage to podocytes impairs the glomerular filtration barrier, resulting in microalbuminuria and proteinuria. These conditions contribute to KD and the progressive decline of renal function [1,9,38].

### 2.3. IR

IR is a major hallmark of pediatric obesity [1,39]. In addition to being a well-documented risk factor for the main cardiometabolic diseases (e.g., type 2 diabetes, metabolic syndrome) [40,41], IR has been found to be closely related to the development of ORG. The characteristic histopathological feature in ORG is represented by podocyte damage [29,32]. Indeed, these cells express both insulin and leptin receptors, which are crucial determinants for their proper function [31]. In the context of obesity, dyslipidemia has been demonstrated to increase leptin and reduce adiponectin levels, triggering a pro-inflammatory immune response (IFN+/IL17+CD4+ T cells) while reducing levels of cells modulating the inflammatory response (Foxp3+CD4+ T cells) [23,42]. At the same time, IR impairs insulin receptor function, directly affecting the integrity of the podocyte barrier by altering the PI3K-Akt-mTOR pathway, which is essential for podocyte adaptive function [23,32]. Moreover, IR is known to be associated with microalbuminuria, because of glomerular hyperfiltration secondary to increased glomerular vascular permeability due to obesity itself [1,12].

The combined effects of RAAS activation, IR, hemodynamic changes, glomerular filtration impairments, lipotoxicity, and the subsequent inflammatory, adipokine dysfunction, and oxidative stress create a vicious cycle with an overproduction of pro-inflammatory cytokines, pro-fibrotic growth factors, and mediators such as tumor necrosis factor (TNF)-alpha, transforming growth factor (TGF)-beta, leptin, and interleukin (IL)-6 favoring endothelial dysfunction and increasing vascular permeability [23,29,32]. Taken together, these mechanisms contribute not only to development of KD but also to its progression [1,2,29].

## 3. KD in Children with Obesity: State of the Art

Despite some sparse and controversial evidence [38,43], the impact of overweight and obesity on pediatric kidney health has been well documented in recent years [8,9,11,44,45,46,47] (Table 1).

In 2012, Vivante et al. examined the correlation between BMI and both diabetic and non-diabetic end-stage renal disease (ESRD) in 1.2 million Israeli adolescents undergoing general health screenings for mandatory military service [8]. Over a 25-year follow-up period, 874 individuals developed ESRD, resulting in an overall incidence rate of 2.87 cases per 100,000 person-years. Children classified as overweight (BMI > 85th percentile) or obese (BMI ≥ 95th percentile) faced a higher risk of developing ESRD, with incidence rates of 6.08 and 13.40 cases per 100,000 person-years, respectively. Both overweight (HR, 5.96; 95% CI, 4.41–8.06) and obesity (HR, 19.37; 95% CI, 14.13–26.55) were identified as significant risk factors for developing both diabetic and non-diabetic ESRD [8].

Additionally, a growing body of evidence supports the complex interplay between obesity and CKD, not only in adults [48] but also in children [46,47,49,50,51]. Increased prevalence of albuminuria, glomerular filtration impairments (both hyperfiltration and reduced eGFR), hypertension [27], dyslipidemia, and hyperinsulinemia have been reported for adolescents with obesity and CKD [45,49].

To date, the evidence on KD in children with obesity remains highly heterogeneous, as studies have examined different clinical features as expressions of KD [1,52,53]. Podocyte injury is widely recognized as a primary factor in organ-related injury (ORG), closely linked to proteinuria in the subnephrotic range (<3.5 g/day in adults and <1000 mg/m^2^/day in children) [1,29] and to gradual KD progression [1,29,49]. Notably, microalbuminuria is a key marker of KD progression. A notable study involving 142 children with obesity categorized by eGFR (normal, decreased, and elevated) characterized the role of microalbuminuria as a CKD marker [9]. The “decreased GFR” group exhibited significantly higher albuminuria levels than those of controls [9]. Furthermore, this group also showed elevated levels of neutrophil gelatinase-associated lipocalin (NGAL) and megalin excretion [9]. Importantly, the “normal GFR” group showed high levels of cholesterol, triglycerides, and serum uric acid, as well as elevated NGAL, which is associated with CKD [9]. Together, these results suggest the potential for persistent albuminuria to serve as an effective marker of organ-related injury and highlight the utility of metabolic biomarkers such as cholesterol, triglycerides, serum uric acid, and NGAL as early indicators of CKD in patients with obesity and normal GFR [9].

Among clinical features of organ-related injury, hypertension also plays a prominent role [27,42]. Children with obesity and metabolic dysfunction often present early elevations in blood pressure (BP) [11,27]. An Israeli study involving 598,702 adolescents evaluated for military eligibility divided participants into high-BMI (>85th percentile) and normal-BMI (<85th percentile) groups, further stratified into four BP risk classes. Groups C (BP of 130/80–139/89 mmHg) and D (BP of ≥140/90 mmHg) were associated with increased early KD risk, particularly among adolescents with high BMI. The authors emphasized the critical role of high BMI and BP values in adolescence as determinants of early KD development in young adulthood [34].

A large American study involving 801,019 children and adolescents aged 3 to 17 years, with a maximum follow-up of 5 years, found a significant increase in hypertension risk among patients with high normal body weight above the 60th percentile of BMI for age. This risk further escalated with weight gain [27]. Given these findings, monitoring BP levels is crucial in managing pediatric obesity to mitigate the risk of KD [50,51]. Notably, strict BP control has shown beneficial effects on kidney function in young patients treated with angiotensin-converting enzyme (ACE) inhibitors [54,55].

Robust evidence highlights hyperuricemia as one of the strongest predictors of pediatric KD [11,16]. A retrospective, cross-sectional study investigated 117 overweight and obese children in nephrology care, classified according to CKD severity (stages I–V based on eGFR). Patients in stages III–V of CKD exhibited higher serum uric acid levels than those in lower stages. Additionally, direct associations were found between uric acid and BMI (r = 0.44; *p* = 0.0001), BMI z-score (r = 0.20; *p* = 0.02), albuminuria (r = 0.31; *p* = 0.0001), and elevated systolic BP (r = 0.24; *p* = 0.004) [16]. Therefore, hyperuricemia serves not only as a predictor of high cardiovascular disease risk but also as a strong marker of KD in childhood, allowing for early identification of at-risk patients [16].

An elegant study examined kidney function on 614 young patients with overweight and obesity undergoing a multidisciplinary lifestyle intervention [56]. The authors demonstrated a positive impact of targeted lifestyle interventions on overall kidney health (mainly expressed as eGFR levels) [56].

In recent years, metabolic phenotypes of pediatric obesity have been extensively studied [57,58,59,60,61,62]. In addition to the well-known cardiometabolic risks associated with the metabolically unhealthy obesity (MUO) phenotype [57,58,59], evidence has shown an unfavorable risk profile in patients with the metabolically healthy obesity (MHO) phenotype [11,56,60,61,62]. A recent Italian study enrolled 396 children with obesity, clustering them into MHO and MUO phenotypes [11]. KD was detected in 20.9% of the cohort, with a higher prevalence in the MUO group than in the MHO group. Notably, HOMA-IR emerged as a key determinant in KD development across both phenotypes, while uric acid was identified as a strong predictor of KD only in the MHO group [11]. In addition to its established role in cardiometabolic risk, the authors emphasized the importance of monitoring uric acid levels in assessing kidney health in patients with MHO [11].

Although still emerging, KD is rapidly becoming a significant obesity-related consequence with serious short- and long-term implications [52,63]. Given this context, early intervention programs are a priority for pediatricians to ensure the best overall care for young patients.

**Table 1:** Main findings of pediatric studies on KD in children with obesity.

Reference	Study Design	Population and Methods	Main Findings
[9]	Case-control study	A total of 142 subjects was enrolled in the study group with the following inclusion criteria: (1) age of 10–16 years; (2) BMI z-score of >2; (3) no arterial hypertension; and (4) signed informed consent. Patients with obesity (BMI z-score > 2) were divided into “elevated GFR” (>130 mL/min/1.73 m^2^ [n = 42]), “normal GFR” (n = 85), and “decreased GFR” (<90 mL/min/1.73 m^2^ [n = 15]) groups according to GFR values estimated by Filler formula. A total of 62 subjects were enrolled as the control group.	Patients with obesity presented with albuminuria, lower serum adiponectin (*p* = 0.005), and higher urine Gal-3 concentrations (*p* = 0.004). Compared to controls, the “Decreased GFR” group showed higher serum uric acid (*p* = 0.004), triglycerides (*p* = 0.037), and cholesterol (*p* = 0.043) levels as well as significantly higher NGAL urine concentration and daily urine megalin excretion (*p* = 0.005). “Normal GFR” obese patients presented a strong correlation between urine Gal-3 concentration and urine NGAL concentration (*p* = 0.001, r = 0.706).
[11]	Cross-sectional study	396 children and adolescents with obesity aged <18 years and a BMI that was >95th percentile according to reference values. Patients were classified according to obesity phenotypes such as MUO and MHO phenotypes. The study population was also stratified based on the presence or absence of KD (defined as the presence of reduced eGFR and/or albuminuria after a 3-month period of confirmation).	KD was found in 20.9% of the study population (25.8% of MUO group; 13.1% of MHO group). Children with KD showed higher BMI-SDS and higher HOMA-IR. MUO patients showed higher prevalence of hypertension, NAFLD and increased HOMA-IR values than those without KD (all *p* < 0.005). MHO patients showed significant differences for SBP-SDS, uric acid, fasting insulin, HOMA-IR, platelets, total cholesterol and low-density lipoprotein cholesterol values. MUO and MHO subjects had respectively an OR to show KD of 1.92 (95% CI: 1.22–3.01; *p* = 0.005) and 1.05 (95% CI: 1.00–1.09; *p* = 0.028) after adjustments. HOMA-IR was closely associated to KD in MUO group (OR = 2.07; 95% CI: 1.20–3.57; *p* = 0.007), while HOMA-IR (OR = 1.15; 95% CI: 1.02–1.29; *p* = 0.011) and uric acid (OR = 1.15; 95% CI: 1.02–1.30; *p* = 0.010) were the only significant risk factors for KD in MHO group.
[16]	Observational cross-sectional study	117 children with CKD classified according to disease severity as stage I–II (eGFR > 60 mL/min/1.73 m^2^) or stage III–V (eGFR < 60 mL/min/1.73 m^2^). Overweight was defined as a BMI of >85th percentile for age and sex, and obesity was defined as a BMI of >95th percentile. Hypertension was defined as SBP of >95th percentile for age, sex, and height or receipt of one or more antihypertensive agents (excluding the use of angiotensin-converting enzyme inhibitors for proteinuria). eGFR was calculated using a modified Schwartz formula.	The prevalence of hyperuricemia in stage III–V was 70%. Children with stage III–V CKD were more likely to have hyperuricemia (OR, 4.6; 95% CI: 2.2–9.4; *p* < 0001). Children with hyperuricemia were more likely to be hypertensive (OR, 2.1; 95% CI: 1–4.1; *p* = 0.03). Hyperuricemia was significantly associated with increased BMI, albuminuria, and renal dysfunction with reduced eGFR and hypertension (all *p* < 0.05). Significant linear relationships between eGFR and urate and between BMI and urate were detected (both *p* = 0.0001).
[27]	Retrospective cohort study	A total of 801,019 youths aged 3 to 17 years (mean [SD] age, 9.4 [4.6] years; 409,167 [51.1%] female]; 391,852 [48.9%] male) were recruited. Baseline sex-specific BMI for age and change in the distance to the median BMI for age during the 5-year follow-up were compared. Cox proportional hazards regression models with age as a time scale were used to assess hypertension risk, adjusted for sex, race, and ethnicity, socioeconomic status, baseline year, and birth year.	Compared with youths with a baseline BMI for age in the 40th to 59th percentiles, the aHR for hypertension within a maximum of 5 years was 1.26 (95% CI: 1.20–1.33) for youths between the 60th and 84th percentiles. With every 1-unit annual increase in the distance to the median BMI for age, the aHR increased by 1.04 (95% CI: 1.04–1.05). The aHR was 4.94 (95% CI: 4.72–5.18) in youths with a baseline BMI for age in the 97th percentile or higher who maintained their body weight. Weight gain increased the risk associated with baseline BMI for age in the 97th percentile or higher with an aHR of 1.04 (95% CI: 1.04–1.05) per 1-unit annual increase in the distance to the median BMI.
[34]	Cohort study	The study population included 598,702 Israeli adolescents (age 16–20 years) evaluated for eligibility for military service. 505,816 (84.5%) were lean (BMI < 85th percentile), and 92,886 (15.5%) had a high BMI (BMI ≥ 85th percentile). According to BP levels, patients were clustered into group A (<120/<80 mm Hg; reference group), group B (120/<80–129/<80 mm Hg), group C (130/80–139/89 mm Hg), and group D (≥140/90 mm Hg). Early KD in young adulthood was defined as albuminuria of ≥30 mg/g with an estimated glomerular filtration rate of ≥60 mL/(min·1.73 m^2^).	Of 598 702 adolescents (54% men), 2004 (0.3%) developed early KD during a mean follow-up of 15.1 (7.2) years. The aHRs for early KD in BP group C were 1.17 (1.03–1.32) and 1.51 (1.22–1.86) among adolescents with lean and high BMIs, respectively. Corresponding HRs for KD in group D were 1.49 (1.15–1.93) and 1.79 (1.35–2.38) among adolescents with lean and high BMIs, respectively.
[38]	Cross-sectional study	A cohort of 1078 youths of both sexes in the range of 11–18 years of age was recruited. Patients were classified based on sex and age BMI percentiles (LMS method) into five BMI groups (as severely thin, thin, healthy, overweight, and obese), and measurements of urinary biomarkers of kidney injury (KIM-1, NGAL, and ACR) were obtained.	The median urinary levels of NGAL, ACR, and particularly KIM-1 (as a more sensitive indicator of kidney injury), showed no significant differences across the BMI groups. Notably, moderate correlations between BMI, KIM-1 and NGAL were identified in severely thin girls.
[46]	Review	7 observational studies (4 prospective cohorts and 3 retrospective cohorts) evaluating the effect of obesity in childhood and adolescence on the occurrence of kidney diseases later in life were included.	Out of 7 cohort studies reported statistically significant positive links between obesity in early life and kidney disease later in life. 5 of the included studies adjusted for hypertension and only 2 adjusted for diabetes as major risk factors for CKD.
[47]	Cross-sectional cohort study	600 children with overweight and obesity were enrolled (mean age was 12.20 ± 3.28 years; mean BMI z-score was 3.31 ± 0.75), of which 53.5% were female. 21.3% of the children were OW, 44.7% OB, and 34.0% with severe OB. eGFR equations and rSCr/Q-age or -/Q-height were assessed.	96.5% of the children had SCr/Q-height and SCr/Q-age within the reference interval (0.67–1.33). SCr/Q-height was weakly inversely associated with BMI z-score (r = −0.109, *p* = 0.007). SCr and SCr/Q-age did not correlate with BMI z-score. SCr/Q-age and nearly all eGFR equations correlated with HOMA-IR and HDL cholesterol, triacylglyceride, serum uric acid, and ALT concentrations. All examined creatinine-based eGFR equations were positively correlated with fat mass and waist-to-hip ratio.
[49]	Review	16 papers examining the prevalence of various biomarkers in adolescents with obesity and CKD were included.	Microalbuminuria was found in 75% of the studies analyzed as the most prevalent biomarker in adolescents. Hypertension was found to be as low as 13.6% and as high as 70.6%. Low HDL-C values were reported with a prevalence range of 1.39% to 20%.
[53]	Cross-sectional study	3118 youth with OW/OB (aged 5–14 years) and 286 healthy normal weight youth. MRGFR was defined as eGFR > 60 and <90 mL/min/1.73 m^2^. eGFR was calculated through eGFRBSE and eGFRFAS.	MRGFR was found in 3.8% in NW vs. 7.8% in OW/OB (*p* = 0.016) by eGFRBSE, and 8.7% in NW versus 19.4% in OW/OB (*p* < 0.0001) by eGFRFAS. MRGFR was associated with an altered CMR profile in youths with OW/OB. The eGFRFAS equation identified a higher prevalence of youth with MRGFR compared to eGFRBSE equation.
[56]	Prospective cohort study	614 children with overweight and obesity (mean age of 12.17 ± 3.28 years, 53.6% female, mean BMI z-score of 3.32 ± 0.75). Of these, 557 patients completed follow-up interventions. SCr was rescaled using Q-age and Q-height polynomials. At baseline, 95–97% of the children had a SCr/Q-height and SCr/Q-age in the normal reference range [0.67–1.33].	SCr/Q significantly increased in every follow-up visit, and linear mixed regression analyses demonstrated slopes between 0.01 and 0.04 (corresponding to eGFR FAS reduction of 1.1–4.1 mL/min/1.73 m^2^) per visit. BMI z-score reduced in both sexes, and this reduction was significantly higher in males (slope = −0.07556, *p* < 0.0001). No correlation between change in rescaled SCr and BMI z-score reduction was demonstrated.

Abbreviations: ACR, albumin creatinine ratio; ALT, alanine aminotransferase; aHR, adjusted hazard ratio; BMI, body mass index; BP, blood pressure; BSA, body surface area; CKD, chronic kidney disease; CMR, cardiometabolic risk; eGFR, estimated glomerular filtration rate; eGFR BSE, eGFR bedside Schwartz equation; eGFR FAS, eGFR full age spectrum equation; Gal-3, galectin-3; NW, normal weight; OB, obese; OW, overweight; HDL-c, high density lipoprotein-cholesterol; HOMA-IR, homeostasis model assessment of insulin resistance; KIM-1, kidney injury molecule-1; KD, kidney damage; KL, kidney length; MRGFR, mildly reduced estimated glomerular filtration rate; MHO, metabolically healthy obesity; MUO, metabolically unhealthy obesity; NAFLD, non-alcoholic fatty liver disease; NGAL, neutrophil gelatinase-associated lipocalin; OR, odds ratio; SBP, systolic blood pressure; SCr, serum creatinine; SD, standard deviation.

## 4. Clinical Implications

The relevant burden of KD on the overall cardiometabolic health of children with obesity poses several clinical and prognostic implications [1,2,3]. Indeed, the coexistence of certain shared pathogenic risk factors such as inflammation, IR, dyslipidemia, and oxidative stress predisposes children with obesity and KD to an increased risk of CKD and cardiometabolic complications (e.g., metabolic syndrome, cardiovascular disease) [1,2,3,8,9].

Since early KD might lead to progressive decline in kidney function [1,3,7], multidisciplinary management including careful and timely monitoring of kidney health is of paramount importance for children with obesity [1,2]. In this framework, the recent position paper of the International Society for Pediatric and Adolescent Diabetes (ISPAD) has recognized KD as an emerging obesity consequence significantly increasing the overall cardiometabolic risk of children with obesity, thereby necessitating early detection and intervention [10]. Starting from 6 years of life, a standard assessment of serum creatinine levels and of microalbuminuria is recommended for all children with obesity at first evaluation [10]. In the case of KD presence, children should be referred to a pediatric nephrologist [2].

Nonetheless, in light of the overall increased cardiometabolic burden of this at-risk young population, a tailored follow-up according to metabolic risk status has been also recently implemented in our daily clinical practice for children with obesity without baseline KD [2]. Indeed, three metabolic risk classes (as “low”, “intermediate”, and “high”) defined on the basis of the presence of family history for cardiometabolic disease and of the presence of steatotic liver and/or metabolic dysfunction [13] provide guidance for a scheduled follow-up of these patients [2]. More specifically, BP measurement and albuminuria evaluation every 12 months and serum creatinine assessment every 24 months in prepubertal patients and yearly in pubertal ones should be performed for patients classified as “low risk”. Patients at “intermediate risk” should undergo BP measurement, biochemical evaluation, and albuminuria evaluation every 12 months, while BP measurement and albuminuria assessment every 3 months and biochemical evaluation every 6 months should be performed for those at “high risk”.

Taken together, regular monitoring of kidney function, lifestyle modifications, and early intervention strategies are crucial in managing the heightened cardiometabolic risk in these young patients.

## 5. Future Directions

Kidneys play a central role in cardiometabolic health, because of their intimate interplay with dysmetabolism and cardiovascular function [1,2]. Therefore, early identification and timely intervention might prevent the progression of KD and its related consequences over time, as supported by the currently available data in the field [1,2,3,8,9]. Therefore, routine screening for kidney health (including BP monitoring, microalbuminuria, eGFR, and metabolic health assessment) should be included as part of the standard clinical practice in managing children with obesity [2,13].

To pave the way for a better knowledge of KD in children with obesity, certain key points should be considered. Based on a deeper pathophysiological understanding of KD (including not only the role of inflammation, oxidative stress, and hormonal changes related to obesity but also the overall impact of the intriguing interplay among obesity, hypertension, and diabetes on kidney health), further research should be focused on the identification of novel biomarkers for KD (both urinary and blood) [64] for earlier intervention programs and routine screening protocols. Moreover, examining genetic predisposition and epigenetic changes in children with obesity might elucidate individual susceptibility to KD.

Longitudinal studies tracking kidney function in children with obesity from early childhood to adolescence are needed to provide novel insights into KD progression and its long-term consequences and identify critical periods for interventions. Indeed, the availability of repeated measures over time can be valuable in enhancing the significance of the analysis, because of the retrospective nature of the most recent evidence.

Thereby, effective tailored lifestyle interventions can prevent or mitigate the risk of KD in this vulnerable population. On these grounds, a multidisciplinary approach (including pediatric nephrologists, endocrinologists, and nutritionists) should be advocated not only to create an optimal standard care model but also to increase medical awareness of obesity-related KD in childhood.

In light of the most recent evidence on the potential usefulness of artificial intelligence (AI) in different medical conditions [65], including renal diseases [66], its use in this context might significantly improve early detection and monitoring of pediatric kidney health. Indeed, implementing AI in analyzing large datasets can help identify patterns and risk factors associated with KD in the context of childhood obesity.

Additional research is also needed for pharmacological interventions. Integrated treatment strategies addressing multiple obesity cardiometabolic consequences are warranted. Moreover, medications targeting inflammation and/or IR might be developed to offer new insightful therapeutic avenues.

Overall, more scientific efforts are essential for a deeper understanding of KD in pediatric obesity to enhance both preventions and treatment strategies.

## 6. Conclusions

Emerging literature highlights the significant but often overlooked risk of KD as an obesity consequence in childhood. Because of the well-recognized role of obesity as a risk factor for CKD development and progression, timely monitoring of kidney health in children with obesity is of paramount importance. Indeed, early detection of KD might contribute to prevention or mitigation of the risk of CKD later in life. On these grounds, a comprehensive approach including regular screening, lifestyle interventions, and targeted treatments to improve not only kidney outcomes but also the overall cardiometabolic health of children with obesity is crucial for this at-risk population. Therefore, continued research in this area is needed to develop effective strategies for prevention and management of KD in the context of pediatric obesity.

## Data Availability

No new data were created or analyzed in this study. Data sharing is not applicable to this article.
